# A retrospective study of gestational weight gain in relation to the Institute of Medicine’s recommendations by maternal body mass index in rural Pennsylvania from 2006 to 2015

**DOI:** 10.1186/s12884-018-1883-1

**Published:** 2018-06-18

**Authors:** Michael L. Power, Melisa L. Lott, A. Dhanya Mackeen, Jessica DiBari, Jay Schulkin

**Affiliations:** 1 Research Department, American College of Obstetricians and Gynecologists, PO Box 96920, Washington, DC 20090-6920 USA; 2 Smithsonian National Zoo and Conservation Biology Institute, Washington, DC USA; 3 Geisinger, Department of Obstetrics and Gynecology, Division of Maternal-Fetal Medicine, Danville, PA USA; 4 Health Resources and Services Administration, Maternal and Child Health Bureau, Office of Epidemiology and Research, Division of Research, Rockville, MD USA; 5 Department of Obstetrics and Gynecology, University of Washington School of Medicine, Seattle, WA USA

**Keywords:** Pregnancy, Obstetrics, Guidelines, Body mass index, Gestational weight gain, Obesity

## Abstract

**Background:**

In 2009, the Institute of Medicine (IOM) published guidance on gestational weight gain (GWG) modified by maternal pre-pregnancy body mass index (BMI). Estimates indicate that less than half of US pregnant women have GWG within recommendations. This study examined GWG from before (2006–2009) and after (2010–2015) the release of the IOM guidance in a rural, non-Hispanic white population to assess the proportion of women with GWG outside of IOM guidance, whether GWG became more likely to be within IOM guidance after 2010, and identify potential maternal factors associated with GWG outside of recommendations.

**Methods:**

We examined GWG in 18,217 term singleton births between 2006 and 2015 in which maternal pre-pregnancy BMI could be calculated from electronic medical records at Geisinger, PA, and a subset of 12,912 births in which weekly GWG in the third trimester could be calculated. The primary outcome was whether GWG was below, within, or above recommendations based on maternal BMI. The relationships between GWG, maternal BMI, parity, age at conception, gestation length, and maternal blood pressure were examined.

**Results:**

GWG declined with increasing maternal BMI, however, more than 50% of overweight and obese women gained above IOM recommendations. About one of five women gained below recommendations (21.3%) with underweight women the most likely to gain below recommendations (33.0%). The proportion of births with usable data increased after 2010, driven by a higher probability of recording maternal weight. However, the proportion of women who gained below, within or above recommendations did not change over the ten years. GWG above recommendations was associated with higher maternal BMI, lower parity, and longer gestation. GWG below recommendations was associated with lower maternal BMI, higher parity, shorter gestation, and younger age at conception. Maternal blood pressure was higher for GWG outside recommendations.

**Conclusions:**

Despite the publication of IOM recommendations in 2009 and an apparent increase in tracking maternal weight after 2010, GWG in this population did not change between 2006 and 2015. A majority of overweight and obese women gained above recommendations. GWG below recommendations continues to occur, and is prevalent among underweight women.

## Background

Gaining an appropriate amount of weight during pregnancy has been shown to positively influence fetal and maternal health during pregnancy, immediately postpartum, and well into the future [1]. Gestational weight gain (GWG) in a woman’s first pregnancy is a significant predictor of her weight gain in a subsequent pregnancy [2]. Excessive GWG is associated with an increased risk for maternal hypertensive disorders and macrosomia for all maternal body mass index (BMI) categories [3].

Two of the Maternal, Infant and Child Health (MICH) objectives listed in Healthy People 2020 are to increase the proportion of women who gain the recommended amount of weight during pregnancy (MICH 13) and to increase the proportion of women delivering a live birth who had a healthy weight prior to pregnancy (MICH 16.5) [4]. In 2009, the Institute of Medicine published revised guidance on GWG that took account of the mother’s pre-pregnancy BMI for the suggested total GWG and weekly weight gain during the second and third trimester [5]. In 2013 the American College of Obstetricians and Gynecologists endorsed the 2009 IOM GWG by maternal BMI recommendations [6].

This retrospective study examines data on GWG in a population of women who received their prenatal health care at Geisinger between 2006 and 2015. The goals of the study are to characterize GWG for singleton term births in this largely non-Hispanic white, rural population, to assess whether there has been any change in the patterns of GWG in this population over the ten-year period in response to the 2009 IOM recommendations, and to identify maternal factors associated with GWG outside of the IOM recommendations.

## Methods

Geisinger is an open integrated health care delivery system that employs roughly 700 physicians across more than 50 clinical practice sites dispersed over 41 largely rural counties in central and northeastern Pennsylvania. Geisinger serves a patient population of approximately 2.5 million people, both through its own health plan and by accepting payment from other payers both public (e.g. Medicaid) and private (e.g. Blue Cross). Geisinger adopted an electronic health record system in 1995, with access provided to patients as well as to health care providers and managers. Geisinger becomes an integrated “medical home” for its patients [7]. The protocols for this project were determined to meet the criteria for exemption by the Geisinger Institutional Review Board. Electronic medical records (EMR) from 35,758 term (gestation length 259–294 days) singleton births at Geisinger between calendar years 2006 and 2015 (01/01.2006–12/31/2015) were examined. Of those 35,758 births, there were 23,555 that had a valid recorded maternal height and weight either from before the pregnancy or within 30 days of conception which could be used to calculate maternal pre-pregnancy BMI. Maternal BMI was categorized as: underweight (BMI < 18.5 kg/m^2^), normal weight (18.5 kg/m^2^ ≤ BMI < 25 kg/m^2^), overweight (25 kg/m^2^ ≤ BMI < 30 kg/m^2^), and obese (BMI ≥ 30 kg/m^2^). Obesity was further categorized as: class I (30 kg/m^2^ ≤ BMI < 35 kg/m^2^), class II (35 kg/m^2^ ≤ BMI < 40 kg/m^2^), and class III (BMI ≥ 40 kg/m^2^). Of those births, 18,217 had a weight recorded both within the first 30 days of pregnancy and within 7 days of parturition (Table 1). These 18,217 singleton term births that occurred between 2006 and 2015 at Geisinger were examined for total GWG. Total GWG was calculated by the difference between the last weight recorded within seven days before birth and the earliest pregnancy weight recorded (within the first thirty days of pregnancy).

**Table 1:** Basic maternal demographic information for the birth data extracted from electronic medical records by year. There were no significant changes in these parameters between 2006 and 2015

Birth year (N)	Non-Hispanic white (%)	Age at conception (years)	Pre-pregnancy BMI^a^	Postpartum BMI (*N* = 12,581)^b^	Systolic blood pressure^c^ (mmHg)	Gestation length (days)
2006 (418)	96.9	26.2 ± 0.3	27.3 ± 0.3	29.2 ± 0.4	113.2/69.6	275.0 ± 0.4
2007 (1224)	96.1	26.6 ± 0.2	27.7 ± 0.2	28.9 ± 0.2	112.9/69.3	275.7 ± 0.2
2008 (1063)	96.6	26.7 ± 0.2	27.9 ± 0.2	29.6 ± 0.3	112.0/68.8	275.1 ± 0.2
2009 (1242)	94.1	26.8 ± 0.2	27.4 ± 0.2	29.1 ± 0.2	112.1/69.0	276.2 ± 0.2
2010 (1103)	95.1	27.1 ± 0.2	27.5 ± 0.2	29.2 ± 0.2	112.6/69.1	276.2 ± 0.2
2011 (2328)	95.1	26.9 ± 0.1	27.8 ± 0.1	29.5 ± 0.2	112.5/68.6	277.1 ± 0.2
2012 (2486)	93.6	26.8 ± 0.1	27.7 ± 0.1	29.2 ± 0.2	113.3/69.4	276.5 ± 0.1
2013 (2640)	94.7	26.8 ± 0.1	28.2 ± 0.1	29.4 ± 0.2	113.5/69.5	276.7 ± 0.1
2014 (2915)	93.4	27.0 ± 0.1	27.8 ± 0.1	29.5 ± 0.2	113.5/68.9	276.7 ± 0.1
2015 (2798)	93.9	27.1 ± 0.1	28.2 ± 0.1	29.7 ± 0.2	113.5/69.6	276.9 ± 0.1
Total (18,217)	94.5	26.9 ± 0.1	27.8 ± 0.1	29.4 ± 0.1	113.1/69.2	276.5 ± 0.1

^a^Calculated from either a pre-pregnancy weight or the earliest prenatal visit weight within 30 days of estimated conception

^b^From the postpartum visit

^c^Calculated from either a pre-pregnancy measurement or a measurement taken at the earliest prenatal visit within 30 days of estimated conception

The beginning of the third trimester was defined to be within 10 days of day 184 of gestation. There were 12,912 births with at least one recorded weight between days 174 and 194 of gestation and within 7 days of parturition. The difference between the last weight recorded within seven days before birth and the weight recorded closest to day 184 of gestation was divided by the number of days between those weights and then multiplied by 7 to calculate the mean weekly weight gain in the third trimester. For each birth the total GWG and the weekly GWG in the third trimester was classified as below IOM recommendations, within IOM recommendations or above IOM recommendations [21] based on the calculated maternal pre-pregnancy BMI.

### Statistical analysis

The data were analyzed using a personal computer-based software package (IBM SPSS 20.0; IBM Corp, Armonk NY). The main dependent variable for analysis was GWG, and the main independent parameters/covariates were maternal pre-pregnancy BMI, maternal pre-pregnancy blood pressure, the year the infant was born, parity, age at conception, presence/absence of certain pregnancy complications (e.g. depression, hypertension, gestational diabetes), and estimated gestation length.

Values for continuous parameters are given as mean ± SEM. Pearson correlation was used to examine relationships between continuous parameters. Linear regression was used to assess changes over the ten-year period in birth complications and cesarean deliveries. Analysis of covariance (ANCOVA) was used to examine factors affecting GWG. Multinomial logistic regression was used to examine factors that would influence GWG to be either under or above recommendations relative to within recommendations (reference category).

## Results

The 18,217 births were divided among 15,326 women, with 12,717 contributing a single birth, 2355 two births, 226 three births, and 28 women contributing four births. Most births were to multiparous women (2010 births to primiparous women, and 1201 births in which maternal parity was not known). The limited demographic information available for the mothers is presented in Table 1. Mean maternal age at conception, pre-pregnancy BMI, postpartum BMI, pre-pregnancy blood pressure, and gestation length did not differ across these years (Table 1). Almost all (94.5%) of the women were non-Hispanic white. The mean age at conception was 26.9 ± 0.1 years (18–49 years old). The mean gestational length was 276.5 ± 0.1 days (259–294 days). Based on BMI, more than half of the women were overweight (25.4%) or obese (31.6%). Few (3.0%) were underweight. The mean number of weights recorded during the prenatal period for these births was 16, with a median of 15 and a mode of 14.

### Patterns over the ten years

Between 2006 and 2010, the percentage of term singleton births that met our criteria for inclusion (i.e. data to calculate the pre-pregnancy maternal BMI and within 7 days of birth) averaged 33.4%. In 2011, the percentage increased to 52.4% and steadily continued to increase, reaching 77.2% in 2015 (Table 2). The increase was driven by a higher proportion of both recorded pre-pregnancy weights or weights within 30 days of estimated conception and weights within seven days of delivery.

**Table 2:** Singleton term births between 2006 and 2015 from electronic medical records at Geisinger. Note: the lower number of births in 2006 derives from the fact that these births were conceived and delivered in 2006, while for later years the number represents all deliveries in that year regardless of year of conception

Year of birth	Births	Births with pre-pregnancy weight	Percent of births with pre-pregnancy weight	Births also with weight within 7 days of parturition	Percent of births also with weight within 7 days of parturition
2006	1470	706	48.0%	418	28.4%
2007	3626	1940	53.5%	1224	33.8%
2008	3249	1639	50.4%	1063	32.7%
2009	3351	1814	54.1%	1242	37.1%
2010	3133	1667	53.2%	1103	35.2%
2011	4440	3023	68.1%	2328	52.4%
2012	4440	3146	70.9%	2486	56.0%
2013	4256	3217	75.6%	2640	62.0%
2014	4170	3355	80.5%	2915	69.9%
2015	3623	3048	84.1%	2798	77.2%
Total	35,758	23,555	65.9%	18,217	50.9%

**Fig. 1:**
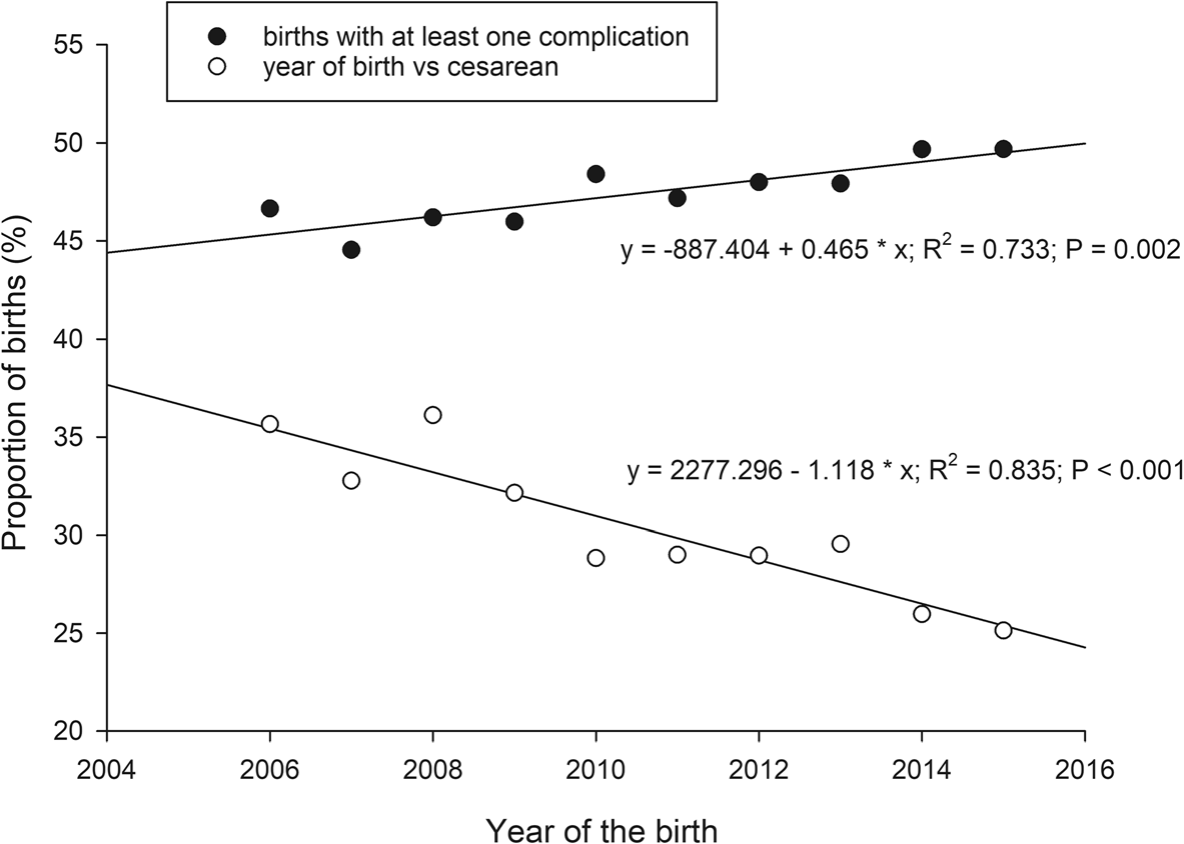
The proportion of term, singleton births with at least one pregnancy complication or with a cesarean delivery from 2006 through 2015

Among the 18,217 births analyzed, 8730 had at least one complication reported (excluding cesarean delivery as a potential complication), leaving 9487 births with no reported complications. There was a small (just under 0.5 percentage points per year) but consistent increase in the proportion of births with at least one recorded complications over the ten years, from about 46% of births before 2009 to 49.7% in years 2014 and 2015 (Fig. 1). Recorded complications included pre-existing hypertension, gestational hypertension, preeclampsia, pre-existing diabetes, gestational diabetes, depression, and polycystic ovarian syndrome. Cesarean delivery accounted for 29% of deliveries. The cesarean rate declined by about 1.1 percentage points per year from above 30% from 2006 through 2009 to 26 and 25% in 2014 and 2015, respectively (Fig. 1).

### Gestational weight gain

Mean total GWG for all 10 years averaged 30.2 ± 0.1 lb. (range = 29.4–31.4 lb). Significant factors affecting GWG were gestation length, pre-pregnancy BMI, pre-pregnancy systolic blood pressure, and parity. The year of birth, maternal age at conception, pre-pregnancy diastolic blood pressure, and complications during pregnancy were not significant factors with respect to GWG. Although significant, the associations between GWG and gestation length (*r* = 0.095, *P* < 0.001) and maternal systolic blood pressure (*r* = − 0.097, P < 0.001) were low. The association with maternal pre-pregnancy BMI was greater (*r* = 0.320, P < 0.001). Parity also appeared to have a larger effect. The estimated marginal means for GWG evaluated at 276 days of gestation, maternal BMI of 28 kg/m^2^, and systolic blood pressure of 113 mmHg were 33.6 ± 0.4 lb., 31.4 ± 0.2 lb., and 27.2 ± 0.2 lb. for the first birth, second birth, and for more than one previous birth, respectively.

Consistent with the positive correlation between gestation length and GWG, the proportion of women who gained above recommendations increased with weeks’ gestation for all BMI categories (Table 3). A majority of overweight women gained above recommendations regardless of gestation length, increasing from 60.6% in the 38th week to 75.2% in the 42nd week (Table 3).

**Table 3:** The proportion of women with GWG above recommendations by maternal BMI class and weeks of completed gestation. Both week of gestation and BMI class were significant factors for GWG above recommendations

Gestational age	All women	Underweight	Normal weight	Overweight	Obese
37^0^–37^6^	667	12	213	212	230
weeks	(47.1%)	(26.7%)	(37.0%)	(60.6%)	(47.1%)
38^0^–38^6^	1247	20	421	394	412
weeks	(47.5%)	(22.5%)	(38.6%)	(61.6%)	(51.2%)
39^0^–39^6^	3577	61	1116	1101	1299
weeks	(51.0%)	(28.6%)	(42.2%)	(63.6%)	(53.5%)
40^0^–40^6^	2876	49	1037	972	818
weeks	(56.0%)	(32.7%)	(48.4%)	(70.1%)	(56.2%)
41^0^–41^6^	1263	20	480	389	374
weeks	(62.4%)	(37.0%)	(56.4%)	(75.2%)	(62.1%)

**Fig. 2:**
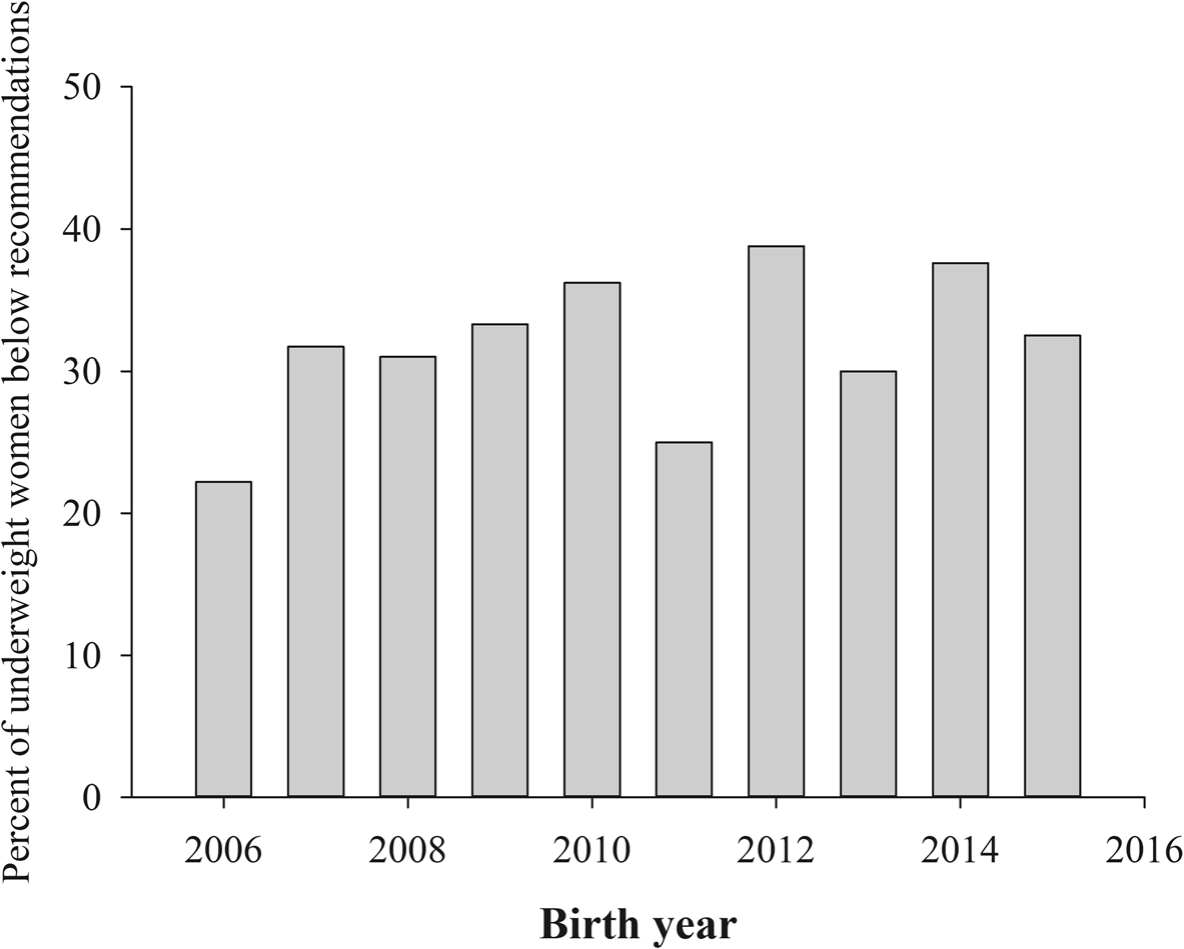
The proportion of underweight women who gained below recommendations did not change over the ten-year period

Although GWG declined with increasing maternal BMI for all births and for births without complications, the proportion of women that gained above the IOM recommendations based on maternal BMI was highest in overweight and obese women (Table 4). These patterns of GWG did not vary over the ten years between 2006 and 2015, with no consistent change in the proportions of women gaining under, within or above IOM recommendations with respect to maternal BMI. Only 25.8% of women in this population gained within recommendations (range = 24.3–27.7%) with an average of 21.3% gaining below recommendations (range = 17.3–24.0%) and 52.9% gaining above (range = 51.4–55.0%). A third of underweight women had a total GWG under IOM recommendations, with no change over these years (Fig. 2).

**Table 4:** Gestational weight gain by BMI class for all term singleton births and for births without complications. Prepregnancy BMI had a significant effect on weight gain, with mean weight gain declining with BMI (*r* = −0.329, P < 0.001 for all births)

	IOM Recommendations (lb)	*N*	Mean GWG ± SEM (lb)	Proportion above IOM recommendations
All births		18,217	30.2 ± 0.1	52.9%
Underweight	28–40	551	35.0 ± 0.5	29.4%
Normal weight	25–35	7305	34.5 ± 0.2	44.7%
Overweight	15–25	4623	32.0 ± 0.2	66.4%
Obese class I	11–20	2815	26.0 ± 0.3	63.0%
Obese class II	11–20	1631	21.1 ± 0.4	50.6%
Obese class III	11–20	1292	17.7 ± 0.5	41.3%
Births with no complications		9487	31.6 ± 0.2	52.8%
Underweight	28–40	340	34.1 ± 0.6	26.5%
Normal weight	25–35	4541	34.7 ± 0.2	44.8%
Overweight	15–25	2461	32.6 ± 0.3	68.0%
Obese class I	11–20	1215	26.8 ± 0.5	65.3%
Obese class II	11–20	596	20.0 ± 0.7	48.2%
Obese class III	11–20	334	17.1 ± 1.1	38.9%

The results were similar for weekly weight gain in the third trimester. Despite a numerical decrease in mean weekly weight gain in obese women, the proportion that gained above recommendations exceeded 60% (Table 5). Underweight women (BMI < 18.5 kg/m^2^) was the only BMI category in which a majority of women did not gain above IOM recommendations. Almost half of underweight women (49.1%) gained below IOM recommendations in the third trimester. Similar to total GWG, there was no change in the pattern of weekly weight gain in the third trimester for under, within or above recommendations across the 10 years, with only 12.9% gaining within recommendations over the ten-year period.

**Table 5:** Weekly weight gain in the third trimester by BMI class for all term singleton births and for births without complications

	IOM Recommendations (lb)	*N*	Mean weekly GWG ± SEM in the third trimester (lb/week)	Proportion above IOM recommendations
All births		12,912	1.06 ± 0.01	64.5%
Underweight	1–1.3	371	1.05 ± 0.03	28.6%
Normal weight	0.8–1.0	5085	1.12 ± 0.01	56.8%
Overweight	0.5–0.7	3230	1.12 ± 0.01	75.9%
Obese class I	0.4–0.6	2028	0.98 ± 0.02	71.8%
Obese class II	0.4–0.6	1230	0.89 ± 0.02	65.0%
Obese class III	0.4–0.6	968	0.90 ± 0.03	64.4%
Births with no complications		6473	1.11 ± 0.01	65.3%
Underweight	1–1.3	220	1.03 ± 0.03	25.9%
Normal weight	0.8–1.0	3098	1.14 ± 0.01	58.0%
Overweight	0.5–0.7	1666	1.17 ± 0.02	79.6%
Obese class I	0.4–0.6	837	1.04 ± 0.03	74.3%
Obese class II	0.4–0.6	420	0.87 ± 0.03	65.5%
Obese class III	0.4–0.6	232	0.96 ± 0.09	63.8%

The multinomial logistic regression results indicated that gestation length, pre-pregnancy BMI, and parity were significant factors for both GWG below and above recommendations, although the effects were not large, except for parity (Table 6). Age at conception was a significant factor for those who were under recommendations, with younger women being more likely to gain under recommendations. Pre-pregnancy systolic blood pressure was significant for those who gained above recommendations, with higher blood pressure associated with a greater likelihood of exceeding GWG recommendations. Both of these effects were small (Table 6). Women who gained within recommendations had both lower pre-pregnancy BMI (26.6 ± 0.1 kg/m^2^; *P* < 0.001) and systolic blood pressure (112.0 ± 0.1 mmHg; P < 0.001) than either those who gained under (28.8 ± 0.1 kg/m^2^ and 113.5 ± 0.2 mmHg) or above recommendations (28.0 ± 0.1 kg/m^2^ and 113.4 ± 0.2 mmHg). Mean gestation length increased by 1.8 days from GWG under recommendations (275.3 ± 0.1 days) to GWG above recommendations (277.1 ± 0.1 days; P < 0.001), a statistically significant, but perhaps not clinically relevant finding. Parity had the largest effect on both gaining above and below recommendations (Table 6). Consistent with the declining estimated marginal means for GWG with increasing parity given above (controlling for gestation length, maternal BMI, and systolic blood pressure), the proportion of women who gained above recommendations consistently declined with parity (62.6, 56.4, and 46.3% for first birth, second birth, and more than one previous birth, respectively; P < 0.001). Conversely, the proportion of GWG below recommendations was lowest for a first birth (14%), almost doubling to 26% for women with two or more previous births.

**Table 6:** Significant factors in the multinomial logistic regression for GWG under, within (reference category) and above IOM recommendations

Parameter	Odds ratio	Standard error	95% confidence interval
Under recommendations versus within recommendations			
Maternal BMI	1.041	0.004	1.034–1.048
Age at conception	0.987	0.005	0.978–0.996
First birth	0.617	0.053	0.521–0.731
Second birth	0.794	0.042	0.716–0.879
Gestation length	0.989	0.003	0.982–0.995
Systolic blood pressure	1.001	0.002	0.998–1.005
Over recommendations versus within recommendations			
Maternal BMI	1.030	0.003	1.024–1.036
Age at conception	0.998	0.004	0.991–1.006
First birth	1.558	0.100	1.374–1.766
Second birth	1.372	0.059	1.261–1.493
Gestation length	1.016	0.003	1.010–1.021
Systolic blood pressure	1.005	0.002	1.001–1.008

## Discussion

The pattern of GWG with respect to pre-pregnancy maternal BMI in this population is concerning. Only 25.8% of women in this population had total GWG within recommendations over the ten years of data, with only 12.9% gaining within recommendations in the third trimester. The majority of women in this population gained above IOM recommendations, especially in the third trimester, in which even a majority of women with a normal pre-pregnancy BMI gained above recommendations. Although overweight and obese women in this population had numerically lower GWG than did women with normal BMI, this reduced GWG still generally exceeded the GWG recommended by IOM, resulting in a large proportion of these women gaining above the IOM recommendations, especially in the third trimester (66.4 and 54.6%, respectively). The only group without a majority gaining above recommendations was underweight women, who disturbingly had a high percentage of GWG below recommendations.

Perhaps most disappointing is that there was no change in these outcomes over the 10 years. Surveys of obstetrician-gynecologists in 2012 and 2014 found widespread knowledge of the IOM recommendations (81.8%) and use of pre-pregnancy BMI to modify GWG recommendations (78.5%) [8]. There were several indications that providers in this study had appropriately counseled their patients regarding recommendations on GWG based on pre-pregnancy BMI. Overweight and obese women did have lower GWG, both total and in the third trimester compared to women with a normal BMI. The fact that women diagnosed with GDM were less likely to have GWG above recommendations potentially represents appropriate practice for these women, with more extensive counseling on diet and GWG. The increase in recorded maternal weights after 2010 indicates a focus on tracking weight in pregnant women and an increased ability to calculate pre-pregnancy and early pregnancy BMI. The fact that the median number of prenatal weights recorded was 15 implies that these women had significant contact with health care providers, and that their pattern of weight gain should have been known to both the women and their care providers. However, despite what appears to be widespread knowledge of the dangers of inappropriate GWG [8] and good tracking of weight during pregnancy, the pattern of inappropriate GWG by most of the women in this population was essentially unchanged over the ten-year period.

Data on mothers with inadequate GWG have unequivocally supported the benefits of increasing total weight gain recommendations for underweight women in reducing the risk of low birth weight babies [9, 10]. It is disconcerting that one third of underweight women gained below IOM recommendations during pregnancy, with almost half gaining below recommendations in the third trimester. Surveys of obstetrician-gynecologists in 2012 and 2014 found they likely underestimated the proportion of their pregnant patients with GWG below recommendations by more than half (7.8%) [8] compared with national data (20%) [11] or the data from this study (21.3%). A concern is that the large proportion of women with GWG above recommendations is resulting in providers failing to recognize women with GWG below recommendations.

Although several maternal factors were significantly associated with GWG outside of recommendations, most (age at conception, gestation length, and systolic blood pressure) had small effects. Even the effect of maternal pre-pregnancy BMI was moderate (Table 6). Parity had a strong effect on the likelihood of gaining an appropriate amount of weight during pregnancy, with the proportion of women gaining above recommendations declining with the number of previous births. This result is consistent with a recent prospective study in Brazil that found that GWG was highest in primiparous women [12]. Unfortunately, the decline of GWG above recommendations with parity did not result in a large increase of women gaining within recommendations. Rather, the proportion of GWG below recommendations increased with parity such that more than one-of-four women with two or more previous births gained below recommendations. Relatively little research appears to have focused on the effect of parity on GWG and how the two factors together might affect differences in health outcomes for mother and child. Higher parity is associated with higher infant birth weight and a greater risk of macrosomia [13]. Children of primiparous mothers have higher body fat at age thirty, and the effect of parity was independent of the effects of maternal BMI and GWG [14]. We suggest that an examination of whether GWG recommendations should be adjusted for maternal parity may be warranted.

The limitations of this study include its retrospective nature, which means the findings are associations and causality cannot be assumed, and the racial homogeneity of the population (94.4% non-Hispanic white), which may limit the extent to which the findings can be generalized to the entire US population. Also, the majority of women were overweight or obese, although, unfortunately, that may be representative of US women of childbearing age. The amount of usable data from the EMR system varied systematically across the years, primarily due to progressively higher incidence of recorded pre-pregnancy and early pregnancy weights after 2010, such that the characterization of GWG is based on less than 40% of term births between 2006 and 2010, and more than 50% of term births after 2010, reaching a maximum of more than 77% of term births in 2015. Thus, GWG is likely more reliably characterized for this population after 2010. However, based on the number of weights taken during pregnancy, the women included in this study were women that had consistent contact with health care providers. They represent women who would be expected to be appropriately counseled and monitored during their pregnancy, regardless of the year the birth occurred, and thus we argue they are comparable across years.

Our expectation was that any effects of the IOM GWG recommendations on patient behavior would be most evident in an integrated system with an extensive medical records system such as Geisinger. The lack of change in GWG across the ten years, despite the general acceptance of the IOM recommendations by obstetrician-gynecologists [8], and the apparent increase in weight tracking after 2010 at Geisinger, reinforces other studies that suggest that obstetrician-gynecologists require additional tools and strategies to encourage behavior change and modify their patients’ GWG effectively [8, 15, 16]. Our data suggest that GWG is resistant to simple interventions, such as increased tracking and recording of weight during pregnancy. A prospective cohort study found little association between receiving provider advice on GWG and gaining within IOM recommendations, suggesting that current provider practice on GWG counseling is not effective [16]. A simple-in-concept health care practice that would improve maternal and neonatal outcomes, helping patients regulate their weight gain during pregnancy within healthy limits, appears to be difficult to accomplish with the current tool set available to obstetrician-gynecologists.

Physicians’ confidence in their ability to affect GWG in their patients was found to be associated with practice effort [8]. If provider counseling on GWG remains ineffective, this could lead to reduced efforts as confidence is lessened by observed lack of change in outcomes. A focus group study found that providers were aware of and concerned about the risks associated with excess GWG, but were concerned that their training was inadequate. A common motivation for participating in the focus groups was “…to find out what other people are doing”, indicating an interest in learning new counseling methods [15]. This suggests the need for new tools to assist providers in communicating the importance of appropriate GWG to their patients and monitoring weight gain throughout pregnancy. Recent randomized trials of life-style modifications have produced encouraging results, resulting in lower GWG in overweight and obese women [17, 18]. Among women who were not low income, consistent GWG tracking was associated with lower GWG [19]. Developing provider tools to guide the management of GWG in patients may improve the quality of care, enhance provider efforts to monitor and manage GWG, and have a positive influence on both maternal and child health outcomes.

## Conclusions

Despite the publication of IOM recommendations in 2009 and an apparent increase in tracking maternal weight after 2010, GWG in this population did not change between 2006 and 2015. The majority of women in this population gained above IOM recommendations, especially in the third trimester, in which even a majority of women with a normal pre-pregnancy BMI gained above recommendations. Overweight and obese women were especially likely to gain above recommendations. Although GWG above recommendations is the most common occurrence, GWG below recommendations continues to occur and is prevalent among underweight women.

## Abbreviations

ANCOVA: Analysis of covariance
BMI: Body mass index
EMR: Electronic medical records
GDM: Gestational diabetes mellitus
GWG: Gestational weight gain
IOM: Institute of Medicine
